# Linker Symmetry Lowering as a Strategy to Tune Electronic Properties in Two‐Dimensional Conductive MOFs

**DOI:** 10.1002/chem.71472

**Published:** 2026-08-03

**Authors:** Bowen Tan, Tianyang Chen, Mingyu Jeon, Julius J. Oppenheim, Mircea Dincă

**Affiliations:** ^1^ Department of Chemistry Massachusetts Institute of Technology Cambridge Massachusetts USA; ^2^ Department of Chemical and Biomolecular Engineering Korea Advanced Institute of Science and Technology (KAIST) Daejeon Republic of Korea; ^3^ Department of Chemistry Princeton University Princeton New Jersey USA

**Keywords:** band gap, metal‐organic framework, semiconductors

## Abstract

Two‐dimensional conductive metal–organic frameworks (2D cMOFs) have largely relied on highly symmetric linkers that give rise to symmetry‐protected electronic degeneracies. Although such symmetry usually promotes extended conjugation and often leads to high electrical conductivity, it can also limit band structure tunability. Here, we demonstrate that deliberate lowering of the linker symmetry is an effective strategy for engineering electronic properties in 2D cMOFs. A reduced‐symmetry C_2v_ catechol linker, hexahydroxylphenanthrene (HHP), was synthesized and incorporated into the 2D cMOF Cu_3_(HHP)_2_. Structural analysis reveals preserved long‐range ordering alongside local symmetry descent relative to higher symmetry analogues. Density functional theory (DFT) calculations show that symmetry lowering lifts electronic degeneracies, leading to modified band dispersion and altered band gap characteristics. Together, these results establish linker symmetry as a fundamental parameter for band structure engineering in 2D cMOFs.

## Graphical abstract

We present a two‐dimensional conductive metal‐organic framework (2D cMOF) constructed from an organic linker with lower symmetry than those commonly used in related systems. The resulting reduction in lattice symmetry lifts band degeneracies, reshapes the electronic structure, and opens a larger band gap. This work establishes symmetry lowering as a powerful strategy for engineering the electronic structures of 2D cMOFs.

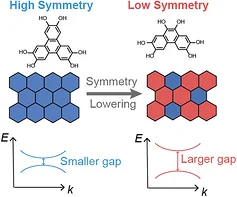

## 1 Introduction

Two‐dimensional π‐conjugated metal–organic frameworks (2D cMOFs) have emerged as a versatile class of materials with broad applications in energy storage and conversion [1, 2, 3, 4], electronics [5], thermoelectrics [6], and chemiresistive sensing [7, 8, 9]. These materials consist of π–conjugated organic ligands bridged by metal nodes to form extended crystalline networks, thereby bridging the gap between discrete molecular electronics and extended band‐like solids. Their electronic structures originate from strong in‐plane π–d conjugation between redox‐active ligands and often square‐planar coordinated transition‐metal centers, coupled with interlayer π–π stacking interactions that enable charge transport along the out‐of‐plane direction [10].

To date, the vast majority of 2D cMOFs have been constructed from highly symmetrical organic linkers, such as hexahydroxytriphenylene (HHTP) and related motifs with D_3h_ symmetry [11]. These linkers typically generate ideal Kagome lattices, which inherently lead to symmetry‐protected band degeneracies. Such degeneracies often result in the formation of Dirac cones or flat bands [12, 13]. Although these features can promote significant band dispersion and facilitate efficient charge transport through high carrier mobility, they simultaneously constrain the accessible electronic states and limit the degree to which the band topology can be tuned. In contrast, symmetry lowering is a well‐established strategy in condensed matter physics to lift degeneracies, split electronic bands, and introduce anisotropy in charge transport [14]. While symmetry breaking has been explored in reticular chemistry to control framework topology or introduce structural complexity [15, 16, 17, 18], its application specifically as a tool for electronic band structure engineering in conductive frameworks remains significantly less explored compared to the traditional focus on highly symmetric building units.

Here, we report the design, synthesis, and characterization of a symmetry‐lowered 2D cMOF, Cu_3_(HHP)_2_, based on the ligand HHP (2,3,6,7,9,10‐hexahydroxyphenanthrene). The HHP linker is a planar catechol‐based ligand with C_2v_ symmetry, representing a symmetry descent from the widely studied HHTP linker. Importantly, HHP retains a comparable electronic structure and redox activity to HHTP, while introducing reduced molecular and electronic symmetry. The resulting Cu_3_(HHP)_2_ framework exhibits crystallinity and structural features analogous to those of the prototypical Cu_3_(HHTP)_2_ system. It thus serves as a unique platform to isolate the effects of symmetry and topology without significantly altering the chemical composition or electronic structure of individual building blocks. We demonstrate that reducing linker symmetry perturbs the lattice symmetry, which in turn relaxes symmetry‐imposed constraints and reshapes the electronic structure. Notably, the accompanying reduction in electrical conductivity places the material in a more tunable semiconducting regime, where charge transport becomes increasingly responsive to external perturbations, making it potentially attractive for applications such as chemiresistive sensing [19, 20]. This work establishes symmetry lowering as a viable and potentially generalizable design principle in cMOFs, offering a new route to engineer band structures and transport properties in 2D materials.

## 2 Results and Discussion

The HHP linker was designed and synthesized through a multistep synthetic route, and its structure was confirmed by ^1^H and ^13^C NMR spectroscopy, as well as high‐resolution mass spectrometry (Figures S1–S3). In contrast to HHTP, which approximates D_3h_ symmetry, HHP adopts a lower C_2v_ symmetry. This symmetry reduction introduces intrinsic anisotropy at the molecular level, breaking electronic degeneracy and providing a foundation for band splitting in the extended solid‐state material. Density functional theory (DFT) calculations further reveal that, despite the distinct molecular geometries of HHP and HHTP, their frontier orbital energies remain comparable, with only modest shifts in both HOMO and LUMO levels (Figures 1a and S4). This electronic similarity is critical, as it minimizes contributions from differences in intrinsic molecular energetics and enables the isolation of topology‐ and symmetry‐driven effects on the electronic structure of the resulting frameworks. Together, these results confirm that HHP preserves the essential electronic characteristics required for constructing 2D cMOFs, while introducing a controlled symmetry perturbation that is expected to modulate band structure and transport properties.

**FIGURE 1:**
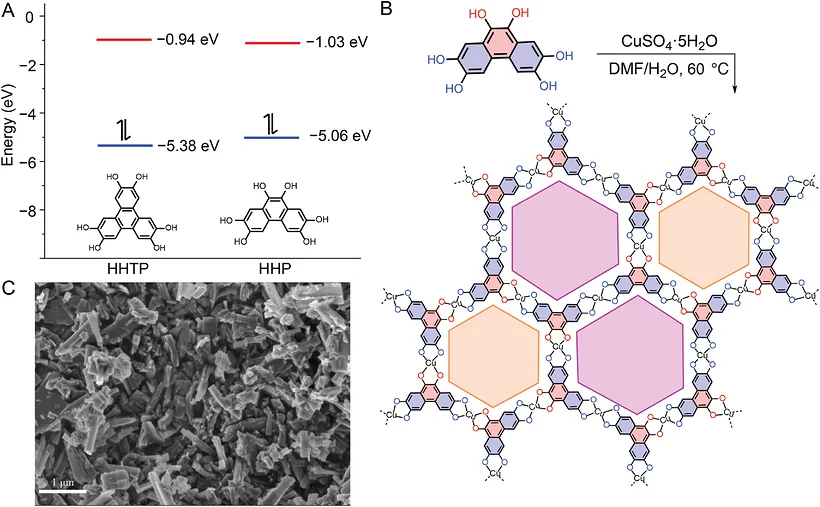
Design and synthesis of Cu_3_(HHP)_2_. (a) Chemical structure and calculated molecular orbitals of HHTP and HHP ligand. (b) Synthetic scheme of Cu_3_(HHP)_2_. (c) Scanning electron microscopy (SEM) image of Cu_3_(HHP)_2_.

A solvothermal reaction between HHP and CuSO_4_·5H_2_O in a water/dimethylformamide (DMF) mixed solvent system yielded Cu_3_(HHP)_2_ as a black powder (Figure 1b). Cu_3_(HHP)_2_ particles have rod‐like morphology measuring approximately 1 µm, as confirmed by scanning electron microscopy (SEM) (Figure 1c). The infrared (IR) spectrum of the product revealed the absence of hydroxyl groups and confirmed the coordination of the linker to the metal center (Figure S5). Pawley refinement of the powder x‐ray diffraction (PXRD) data (Figure 2a) is best described by a hexagonal lattice with unit parameters of *a* = *b* = 33.94 and *c* = 3.32 Å. High‐resolution cryogenic transmission electron microscopy (cryo‐TEM) further supports the structural model, displaying well‐defined hexagonal lattice features, although defects and disordered regions are also present (Figure 2c). Imaging perpendicular to the z‐axis (Figure 2d) reveals contrast fringes aligned approximately parallel to the direction normal to the 2D sheets. The fast Fourier transform (FFT) shows a fringe periodicity of 1.7 nm (Figure 2d inset), which agrees well with the unit cell parameter. Structural modelling reveals two inequivalent pore environments, reflecting local symmetry perturbation within the 2D network. Specifically, Cu_3_(HHP)_2_ features two types of pores: a smaller, regular hexagonal pore and a larger pore with three elongated laterals. Thermogravimetric analysis (TGA) of Cu_3_(HHP)_2_ showed weakly bound or pore‐confined water are largely removed below ∼80°C, followed by significant weight loss above 200°C due to the decomposition of the framework (Figure S6). The nitrogen (N_2_) sorption isotherm of Cu_3_(HHP)_2_ shows a Brunauer–Emmett–Teller (BET) surface area of approximately 407 m^2^/g (Figure S7), which indicates that the material is intrinsically porous and matches the calculated value for the model structure (479 m^2^/g). More importantly, pore size distribution analysis derived from the N_2_ sorption data reveals two pore‐size populations centered at approximately 1.27 and 1.57 nm, experimentally supporting the presence of two distinct pore environments in the framework. Thus, symmetrical reduction occurs locally without compromising global crystallinity, enabling controlled perturbation of electronic structure.

**FIGURE 2:**
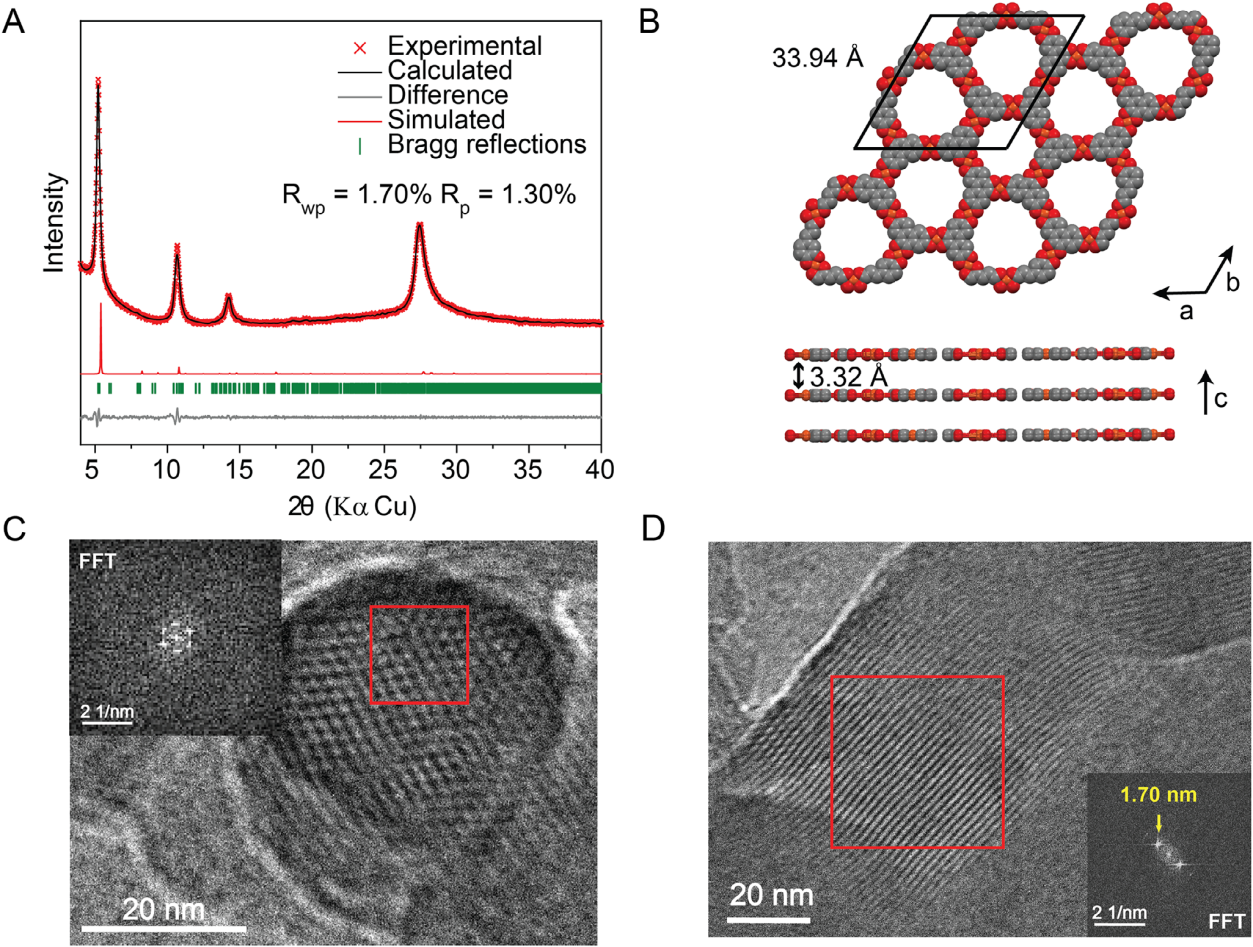
Structure characterization of Cu_3_(HHP)_2_. (a) PXRD patterns of Cu_3_(HHP)_2_ and the modeled structure. (b) Modeled structure of Cu_3_(HHP)_2_. (c,d) Cryo‐HRTEM image of Cu_3_(HHP)_2_ (inset: FFT).

X‐ray photoelectron spectroscopy (XPS) and elemental analysis (EA) were used to determine the chemical composition of Cu_3_(HHP)_2_. The XPS survey spectrum revealed the presence of C, H, O, and Cu, aligning with the expected composition. High‐resolution Cu 2p spectrum of Cu_3_(HHP)_2_ indicated a Cu^2+^/Cu^+^ mixed valence state, as verified by the two peaks at 934.8 eV (Cu^2+^) and 933.1 eV (Cu^+^) after deconvolution (Figure S8). By fitting these peaks, a Cu^2+/^Cu^+^ ratio of 1.33 (57%/43%) was determined. The EA results suggested a chemical formula of Cu_3_(HHP)_2_(H_2_O)_11._ The water molecules are likely either coordinated to the Cu centers or reside as guests within the pores of the framework. We note that neither XPS nor EA analysis provided evidence for nitrogen in Cu_3_(HHP)_2_, suggesting that the framework is indeed neutral and there are no cations in the pores, as is sometimes inferred via decomposition of DMF.

The XPS and EA data suggest that each HHP unit carries approximately 2.3 negative charges, supporting a formula whereby Cu_3_(HHP)_2_ contains a mixture of HHP^2–^ and HHP^3–^ species. The electron paramagnetic resonance (EPR) spectrum of Cu_3_(HHP)_2_ taken at room temperature exhibits a predominant signal attributed to Cu^2+^ with *g* = 2.146 (Figure S9), a typical value for square‐planar Cu^2+^ compounds undergoing rapid spin relaxation [21]. Additionally, a small peak was observed with a *g* value of 2.014, indicating the presence of organic radicals incorporated in the framework [22]. This observation aligns well with the HHP ligands in HHP^3–^ oxidation state, which would carry a persistent organic radical.

Diffuse‐reflectance (DR) optical spectroscopy was employed to gain deeper insight into the electronic structure of Cu_3_(HHP)_2_. DRUV‐Vis‐NIR spectra revealed several peaks from 200 to 400 nm, a broad peak at approximately 600 nm, and intense absorption spanning the entire NIR region (700∼1700 nm) (Figure 3a). The peaks observed in the UV region are attributed to ligand‐centered electronic transitions. The broad peak around 600 nm likely arises from charge transfer between redox‐active Cu centers and HHP linkers. The pronounced absorption in the NIR region is a result of significant in‐plane electronic delocalization through π–d conjugation between HHP ligands and Cu centers, as well as strong interlayer electronic coupling [23]. Fitting the DRUV‐Vis‐NIR data to a Tauc plot revealed a moderate optical gap of 0.87 eV, higher than that reported for Cu_3_(HHTP)_2_ (0.6 eV) (Figure 3a inset) [24]. This increase in optical gap is consistent with our design hypothesis that reducing the linker symmetry in Cu_3_(HHP)_2_ perturbs the electronic structure, resulting in a widened band gap.

**FIGURE 3:**
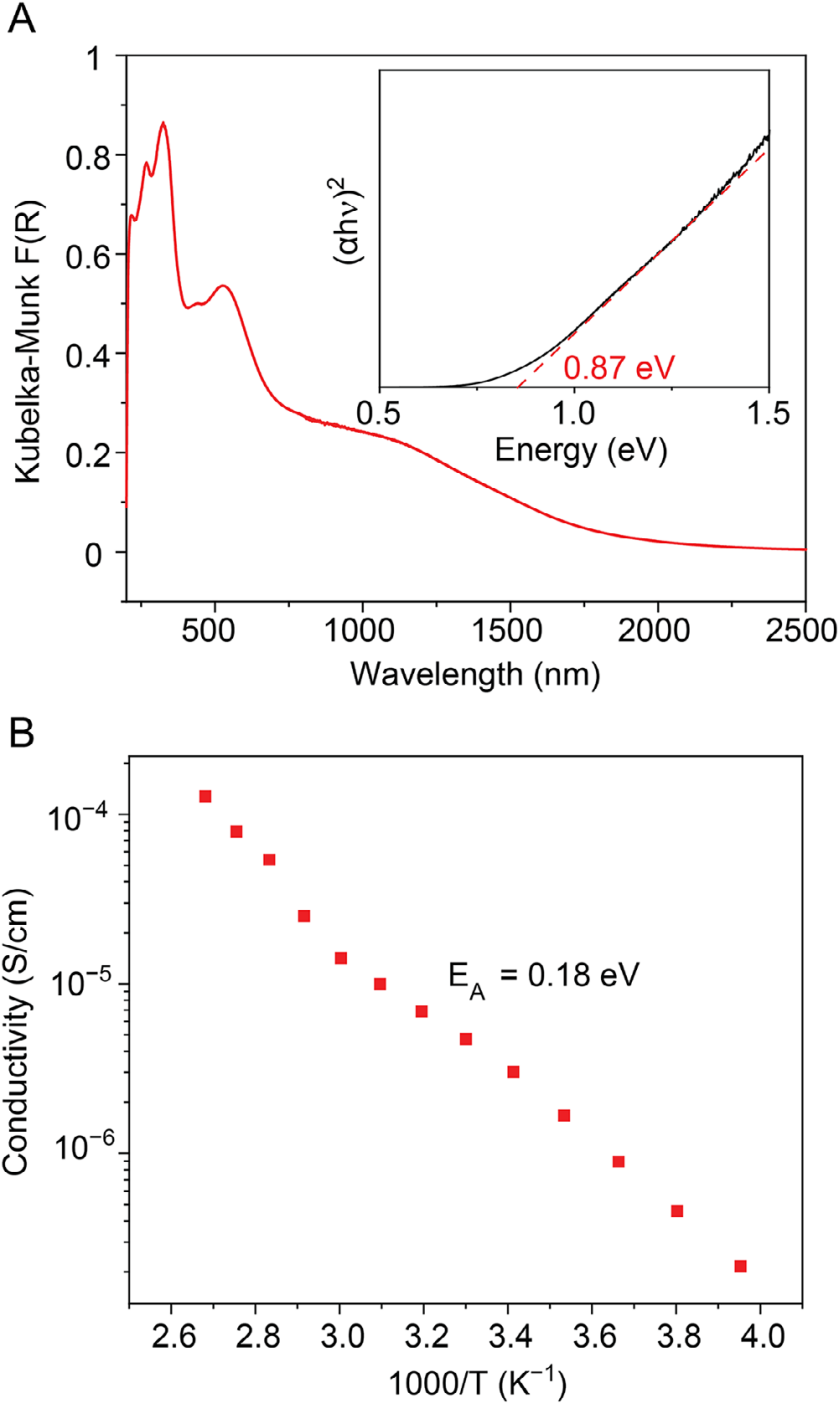
Electronic characterization of Cu_3_(HHP)_2_. (a) DRUV‐Vis‐NIR spectrum of Cu_3_(HHP)_2_ (Inset: Tauc plot). (b) Variable temperature conductivity of Cu_3_(HHP)_2_.

Cu_3_(HHP)_2_ exhibits a room temperature bulk electrical conductivity of (2.46 ± 0.85) × 10^−6^ S/cm, as determined from four‐probe pressed pellet conductivity measurements from multiple different batches (Figure S10 and Table S1). This conductivity value is several orders of magnitude lower than that of Cu_3_(HHTP)_2_, for which we measured a conductivity of 0.0072 S/cm under identical conditions. Notably, this value is consistent with previously reported electrical conductivities of Cu_3_(HHTP)_2_, which typically lie in the range of 0.003∼0.1 S/cm under comparable conditions (Table S2) [25, 26, 27, 28, 29, 30, 31, 32, 33, 34]. Variable‐temperature (VT) conductivity measurements reveal that Cu_3_(HHP)_2_ shows thermally activated transport behavior. Fitting the VT conductivity data to an Arrhenius model yields an activation energy of 0.18 eV (Figure 3b). The activation energies represent the hopping energy barriers according to the nearest‐neighbor hopping mechanism [35]. The reduced conductivity in Cu_3_(HHP)_2_ indicates that lowering linker symmetry perturbs the electronic structure and disrupts efficient charge transport pathways. In particular, symmetry breaking lifts band degeneracies, leading to band splitting and reduced orbital equivalence across the lattice. Taken together, these results demonstrate that linker symmetry plays a critical role in governing the electronic structure and charge transport properties of 2D cMOFs, and that symmetry lowering provides a powerful handle to modulate these properties in a controlled manner.

Although Cu_3_(HHP)_2_ exhibits a lower electrical conductivity than its high‐symmetry analogue, this reduced conductivity places the material in a semiconducting regime relevant for device applications. In contrast to highly conductive or metallic systems, moderate conductivity enables greater tunability of charge transport through external stimuli such as chemical doping or electrochemical bias. This is particularly advantageous for applications such as chemiresistive sensing, where lower baseline conductivity can amplify relative signal change. Thus, the semiconducting nature of Cu_3_(HHP)_2_ provides a functional advantage by enabling responsive and tunable electronic behavior.

To elucidate the electronic consequences of linker desymmetrization, periodic DFT calculations were conducted for Cu_3_(HHP)_2_ and benchmarked against the parent Cu_3_(HHTP)_2_ system using the exact same method (Figure 4). Although both frameworks exhibit characteristic metallic behavior in the out‐of‐plane direction, as evidenced by a band crossing the Fermi level along the A‐to‐Γ path, their in‐plane electronic structures feature distinct semiconducting gaps. Notably, Cu_3_(HHP)_2_ displays a significantly larger in‐plane bandgap than its highly symmetric counterpart. The calculated difference in their bandgaps (ΔE_cal_ = 0.26 eV) is in excellent agreement with the difference in their optical gaps derived from experimental DRUV‐Vis‐NIR spectroscopy (ΔE_opt_ = 0.27 eV). This result strongly substantiates our central hypothesis: transitioning to a lower‐symmetry lattice induces a pronounced lifting of symmetry‐protected band degeneracies. Ultimately, these results establish a rigorous structure‐symmetry‐electronic correlation, demonstrating that deliberate linker symmetry engineering can effectively tune the frontier orbital landscape in 2D cMOFs.

**FIGURE 4:**
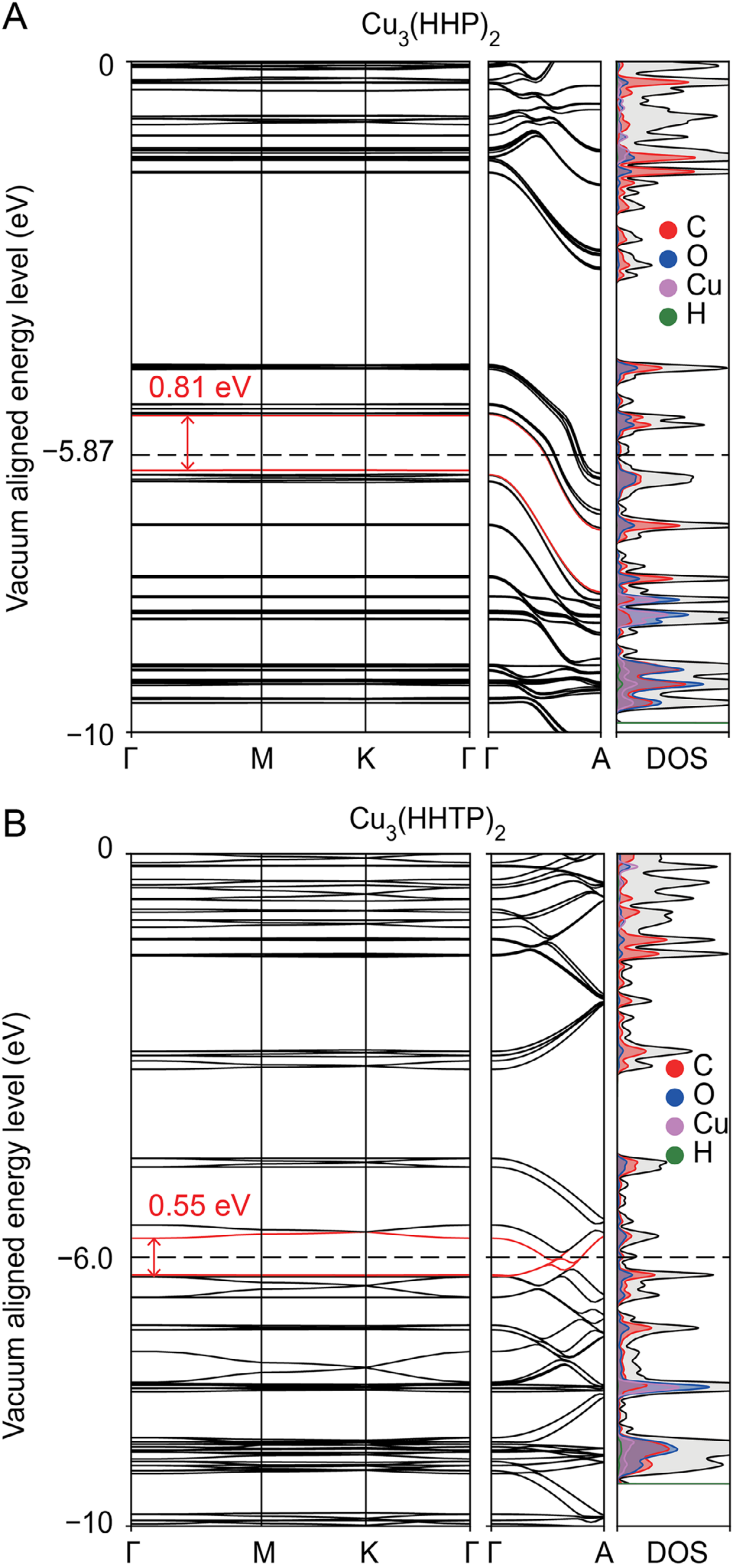
Density functional theory (DFT) calculated electronic band structure and density of states (DOS) of (a) Cu_3_(HHP)_2_ and (b) Cu_3_(HHTP)_2_. The band structures were mapped along the Γ‐M‐K‐Γ (in‐plane) and Γ‐A (out‐of‐plane) paths to capture the full dimensionality of the transport.

## 3 Conclusions

We demonstrate that linker symmetry lowering provides a rational strategy to engineer electronic band structure in two‐dimensional conductive metal–organic frameworks, although conductivity is also influenced by other factors such as crystallinity, oxidation state, stacking, and defects. By incorporating a C_2v_‐symmetric catechol linker into Cu_3_(HHP)_2_, we introduce local lattice distortion while preserving crystallinity. DFT calculations reveal a significantly higher band gap relative to the high‐symmetry analogue Cu_3_(HHTP)_2_. This work establishes symmetry engineering as a fundamental design principle in conductive MOFs and provides a conceptual bridge between reticular chemistry and electronic band theory.

## Conflicts of Interest

The authors declare no conflicts of interest.

## Supporting information




**Supporting File 1**: chem71472‐sup‐0001‐SuppMat.pdf
The authors have cited additional references within the Supporting Information [36, 37, 38, 39, 40, 41, 42, 43, 44, 45].

## Data Availability

The data that support the findings of this study are available in the Supporting Information of this article.
