# Supplementary material for: Linker Symmetry Lowering as a Strategy to Tune Electronic Properties in Two‐Dimensional Conductive MOFs
Source: Chemistry. 2026 Aug 3;32(40):e71472. doi: 10.1002/chem.71472 (PMC13716027; doi:10.1002/chem.71472)
Supplement: Supplementary file 1 [file CHEM-32-e71472-s001.pdf]

Supplementary Information

Linker Symmetry Lowering as a Strategy to Tune Electronic Properties in Two-Dimensional Conductive MOFsBowen Tan[1], Tianyang Chen[1], Mingyu Jeon[1,2], Julius J Oppenheim[3], Mircea Dincă[3]*

[1]Department of Chemistry, Massachusetts Institute of Technology, Cambridge, MA 02139, United States

[2]Department of Chemical and Biomolecular Engineering, Korea Advanced Institute of Science and Technology (KAIST), Daejeon 34141, Republic of Korea

[3]Department of Chemistry, Princeton University, Frick Laboratory, Washington Rd, Princeton, New Jersey 08544, United States

*Corresponding author. Email: mdinca@princeton.edu

Materials and methods

Materials

1,2-bis(3,4-dimethoxyphenyl)ethane-1,2-dione (Ambeed), $TiCl_4$ (Sigma Aldrich), $SnCl_2$ (Alfa Aesar), molybdenum pentachloride (Sigma Aldrich), BBr_3 (Sigma Aldrich), $CuSO_4{\cdot}5H_2O$ (Sigma Aldrich) were used as received without further purification. Dimethylacetamide (DMA), dichloromethane (DCM), acetone, and methanol were used as received without further purification.

Nuclear Magnetic Resonance (NMR)

^{1}H and ^{13}C NMR spectroscopy were recorded on Bruker AVANCE III spectrometers (500 MHz). All chemical shifts are reported in parts per million (ppm). DMSO-d6 was used as the reference for ^{1}H NMR and ^{13}C NMR chemical shifts.

Attenuated total reflectance infrared spectroscopy (ATR-IR)

ATR-IR spectra were recorded in a nitrogen-filled glovebox on a Bruker Alpha FTIR spectrometer equipped with an attenuated total reflectance accessory.

Powder X-ray Diffraction (PXRD)

Laboratory PXRD patterns were recorded using a Bruker Advance II diffractometer equipped with a θ/2θ reflection geometry and Ni-filtered Cu Kα radiation (Kα1 = 1.5406 Å, Kα2 = 1.5444 Å, Kα2/ Kα1 = 0.5). The tube voltage and current were 40 kV and 40 mA, respectively. Samples for PXRD were prepared by placing a thin layer of the appropriate material on a zero-background silicon crystal plate.

N_2 Adsorption-desorption Analysis

The nitrogen adsorption measurements were performed on a Micromeritics ASAP 2020 Surface Area and Porosity Analyzer. An oven-dried sample tube equipped with a TranSeal^{TM} (Micromeritics) was evacuated and tared. The sample was transferred to the sample tube, which was then capped with a TranSeal^{TM}. The sample was activated under a dynamic vacuum of 4 mTorr until the outgas rate was less than 2 mTorr/minute. The evacuated sample tube was weighed again and the sample mass was determined by subtracting the mass of the previously tared tube. The N_2 isotherm was measured using a liquid nitrogen bath (77 K). Ultrahigh purity grade (99.999% purity) N_2, oil-free valves and gas regulators were used for all the free space correction and measurement. Fits to the Brunauer-Emmett-Teller (BET) equation satisfied the published consistency criteria.

Thermogravimetric analysis (TGA)

TGA was performed on a TA Instruments Q500 Thermogravimetric Analyzer at a heating rate of 2.0 °C/min under N_2 gas flow of 5 mL/min on a platinum pan from room temperature to 800 °C.

Elemental analyses (EA)

EA were performed by Robertson Microlit Laboratories, Ledgewood, New Jersey.

Scanning electron microscopy (SEM)

SEM was conducted at MIT MRSEC on a Zeiss Merlin high-resolution scanning electron microscope with an InLens detector at an operating voltage range between 1 and 4 kV.

Cryogenic high-resolution transmission electron microscopy (cryo-HRTEM)

Cryo-HRTEM images were obtained at the Automated Cryogenic Electron Microscopy Facility in MIT.nano on a Talos Arctica G2 microscope operated at an accelerating voltage of 200 kV with a Falcon3EC direct electron detector. Samples were prepared by sonicating MOFs powders in isopropanol for 5 to 10 seconds. Specimens were prepared by drop-casting sonicated samples onto C-flat™ Cu grids with holey carbon for Cryo-EM. All image acquisition was done using EPU at an exposure time of 1 s, with focusing done adjacent to the region imaged to minimize beam exposure prior to image acquisition (standard low dose imaging protocols). Analysis of the raw HRTEM data was done using Gatan Microscopy Suite software (GMS 3).

Electrical conductivity measurements

The measurements were carried out in ambient atmosphere on pressed pellets using a 4-probe probe setup. Pressed pellets of MOF samples were made by adding MOF powders into a 10 mm inner-diameter trapezoidal split sleeve pressing die (Across International) and pressing the die set by a hydraulic pump (MTI corporation) for 5 min. The applied pressure was approximately 1 GPa. The pellet thicknesses were measured after the measurement using a micrometer (Mitutoyo). The resulting pellet was cut into a cuboid by a blade, and placed it onto a piece of dry glass slide with thermally conductive S6 and electrically insulating grease (DuPont Krytox). Four parallel copper wire contacts were attached to the cuboid by carbon paste. The other end of the copper wire was pasted onto the glass slide by carbon paste. The device was mounted onto the sample chuck of a probe station (Janis Cryogenics ST-500) equipped with four micro-manipulators that allowed us to control the position of the probes with a resolution of 5 μm. Electrical contacts were made by gently pressing gold-plated tungsten probes (Janis 7B-100G) into the carbon paste that was on the glass slide. Probes were connected to a source meter (Keithley model 2450) through triax cables (Keithley model

7078-TRX-10). The chamber of the probe station was covered by a stainless-steel lid. This lid had a quartz window in the center, which was covered by a black plastic to keep the device in the dark. Linear I–V curves were obtained by supplying the current and measuring the voltage. The current was duel-swept through the outer two probes, while voltage was measured through the inner two probes. The specific lengths of the conduction channel were measured by a Leica S6 E stereoscopic microscope and calibrated by an external ruler of known distances.

The temperature of the device was balanced by the heater of the probe station chuck and the environment, and was controlled by a temperature controller (Scientific Instrument model 9700). The activation energy Ea was calculated using the equation:

$$\sigma_{e,T} = \sigma_{e,\infty} exp\left(-\frac{E_A}{k_B T}\right)$$

where E_A was extracted from the linear least squares fit of $ln\sigma$ vs. 1000/T. The slope = –(1000*E_A/k_B), where E_A is the activation energy and k_B is the Boltzmann constant.

X-ray photoelectron spectroscopy (XPS)

XPS measurements were performed at the MIT MRSEC using a Physical Electronics PHI Versaprobe II X-ray photoelectron spectrometer equipped with a monochromatic Al anode X-ray source. The main chamber pressure was in the 10^{-10} Torr range. MOFs powder samples were pressed on copper tapes with full coverage. Survey spectra were collected from 0~1100 eV in binding energy (BE), with a resolution of 0.8 eV. High resolution spectra of Cu2p regions were collected with a resolution of 0.1 eV. BE calibration was carried out by shifting the adventitious carbon C1s peak to 284.8 eV. Deconvolution of the C1s spectra were conducted before BE calibration to find out the adventitious carbon C1s peak, given that the MOF samples also contain significant amount of carbon. Gaussian-Lorentzian product function with 30% of Lorentzian component was used for the line shapes during fitting, which is commonly used for C1s.

Computational Methods

The overall methods follow our previous work.[1] To elucidate the electronic consequences of linker symmetry reduction, first-principles calculations were performed within the density functional theory (DFT) framework using the VASP 5.4.1 package.[2] The $Cu_3(HHP)_2$ framework was initialized from experimentally refined coordinates and fully relaxed to a stable hexagonal configuration (a = 32.7558 Å, b = 32.7487 Å, c = 3.3670 Å; alpha = beta = 90.0 degrees, gamma = 120.0469 degrees), while the reference $Cu_3(HHTP)_2$ structure was adopted from a

previous study.[3] Electron-ion interactions were described by the projector augmented-wave (PAW) method,[4,5] with the Perdew-Burke-Ernzerhof (PBE) functional utilized for exchange-correlation effects.[6] Long-range van der Waals interactions were accounted for via Grimme DFT-D3 dispersion corrections.[7] To address the localized d-electrons of the Cu centers, a Hubbard U parameter of 4.0 eV for the Cu centers was applied during geometry optimization and self-consistent field (SCF) calculation.[8] Wavefunctions were expanded with a kinetic energy cutoff of 520 eV, with a SCF convergence threshold of 10^{-5} eV. Structural optimizations were conducted until the interatomic forces were reduced below 0.005 eV/Å. We employed a Gamma-centered 1x1x3 k-point grid for geometry optimization, which was further refined to a 4x4x6 mesh for SCF cycles to obtain a high-fidelity charge density.

Crucially, to resolve the lifting of band degeneracies induced by symmetry lowering, electronic structures were refined using the HSE06 hybrid functional with 20% exact Hartree-Fock exchange. The band structures were mapped along the Gamma-M-K-Gamma (in-plane) and Gamma-A (out-of-plane) paths to capture the full dimensionality of the transport. The density of states (DOS) was evaluated by integrating over the Brillouin zone using weighted k-points along both in-plane and out-of-plane paths. To ensure a rigorous comparison between the two systems, band energy levels were aligned to the internal vacuum level, defined as the electrostatic potential plateau at the center of the framework's pores, using the macrodensity module.[9]

Material Synthesis:

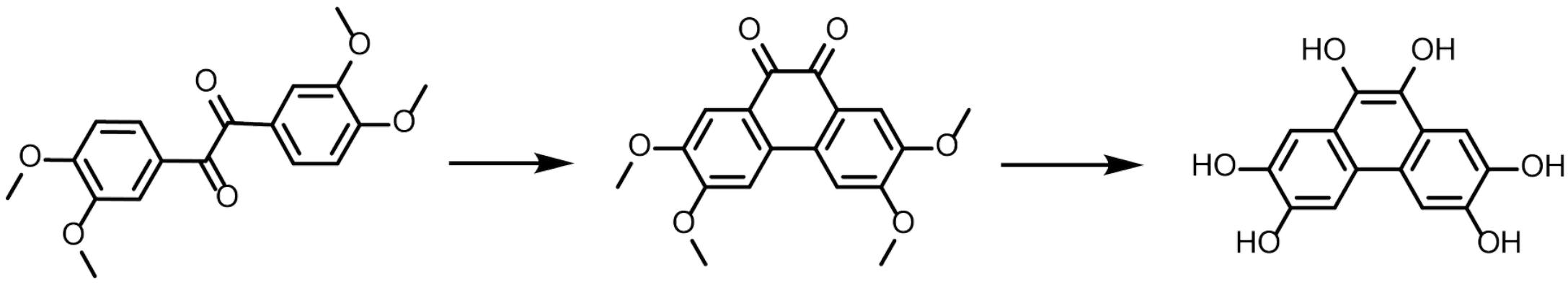

1 2 3

2,3,6,7-tetramethoxyphenanthrene-9,10-dione (2)

Compound 2 was synthesized according the published procedure.[10] A solution of compound 1 (0.25 mmol) in dichloromethane was treated at 0 °C under an argon atmosphere with a solution of $TiCl_4$ in dichloromethane (630 μL, 0.55 mmol, 10 vol%) and stirred for 1 min. Molybdenum pentachloride (151 mg, 0.55 mmol) was then added, and the reaction mixture was stirred at 0 °C for 4 min. An ice-cold saturated aqueous sodium bicarbonate solution (10 mL) was added, and the resulting mixture was stirred at 0 °C for 2 min. The slurry was filtered, and the residual solid was washed thoroughly with dichloromethane (4 × 50 mL). The organic layer was separated, dried over magnesium sulfate, and concentrated under reduced pressure. The crude product was adsorbed onto silica gel and purified by flash column chromatography using dichloromethane/methanol (50:1) as eluent to afford 2 as a golden solid. (yield: 58%). The NMR data are consistent with published results.

2,3,6,7,9,10-hexahydroxyphenanthrene (3)

In a nitrogen-filled Schlenk tube, compound 2 (280 mg, 0.85 mmol) was dissolved in 2 mL of dichloromethane (DCM) to afford a dark red solution. The reaction mixture was cooled to 0 °C, followed by the slow addition of BBr_3 (1.3 mL, 1.44 mmol), upon which the solution rapidly turned purple. The mixture was then allowed to warm to room temperature and stirred overnight. Subsequently, ice-cold water was added, and the mixture was stirred for an additional hour, yielding a green suspension. The resulting solid was collected by filtration to afford a green powder. This solid was transferred to a 25 mL Schlenk flask equipped with a magnetic stir bar, and concentrated HCl (5 mL) was added under a nitrogen atmosphere. $SnCl_2$ (500 mg, 2.21 mmol) was then introduced, and the reaction mixture was stirred at room temperature for 24 h. After completion, the Schlenk flask was transferred into a nitrogen-filled glovebox. The mixture was filtered and the solvent removed under reduced pressure to afford compound 3 as a light gray solid (73% yield). ^1H NMR (25 °C, 500 MHz, DMSO-d6) δ = 9.14 (s, 2H), 9.07 (s, 2H), 8.15 (s, 2H), 7.55 (s, 2H), 7.33 (s, 2H). ^{13}C NMR (500 MHz, DMSO): δ =

145.06, 143.95, 132.09, 120.91, 119.52, 106.61, 105.87 ppm.

$Cu_3(HHP)_2$ (HHP = 2,3,6,7,9,10-hexahydroxyphenanthrene)

A solution of 22.4 mg $CuSO_4 \cdot 5H_2O$ (0.045 mmol) in 2.5 ml H_2O and were added to a solution of 10 mg HHP (0.018 mmol) dissolved in 1 ml DMA and 6 ml H_2O in a 20 mL vial. The vial was heated to 80 °C for 12 hours without stirring. The product precipitated out as black powder. The product was centrifuged, decanted and washed with deionized water twice and acetone twice. The product was dried under vacuum at room temperature. Elemental analysis: (calculated, found for $Cu_3(C_{14}H_3O_6)_2(H_2O)_{11}$), C% (36.11, 36.47), H% (3.01, 3.09), N% (0, < 0.10%).

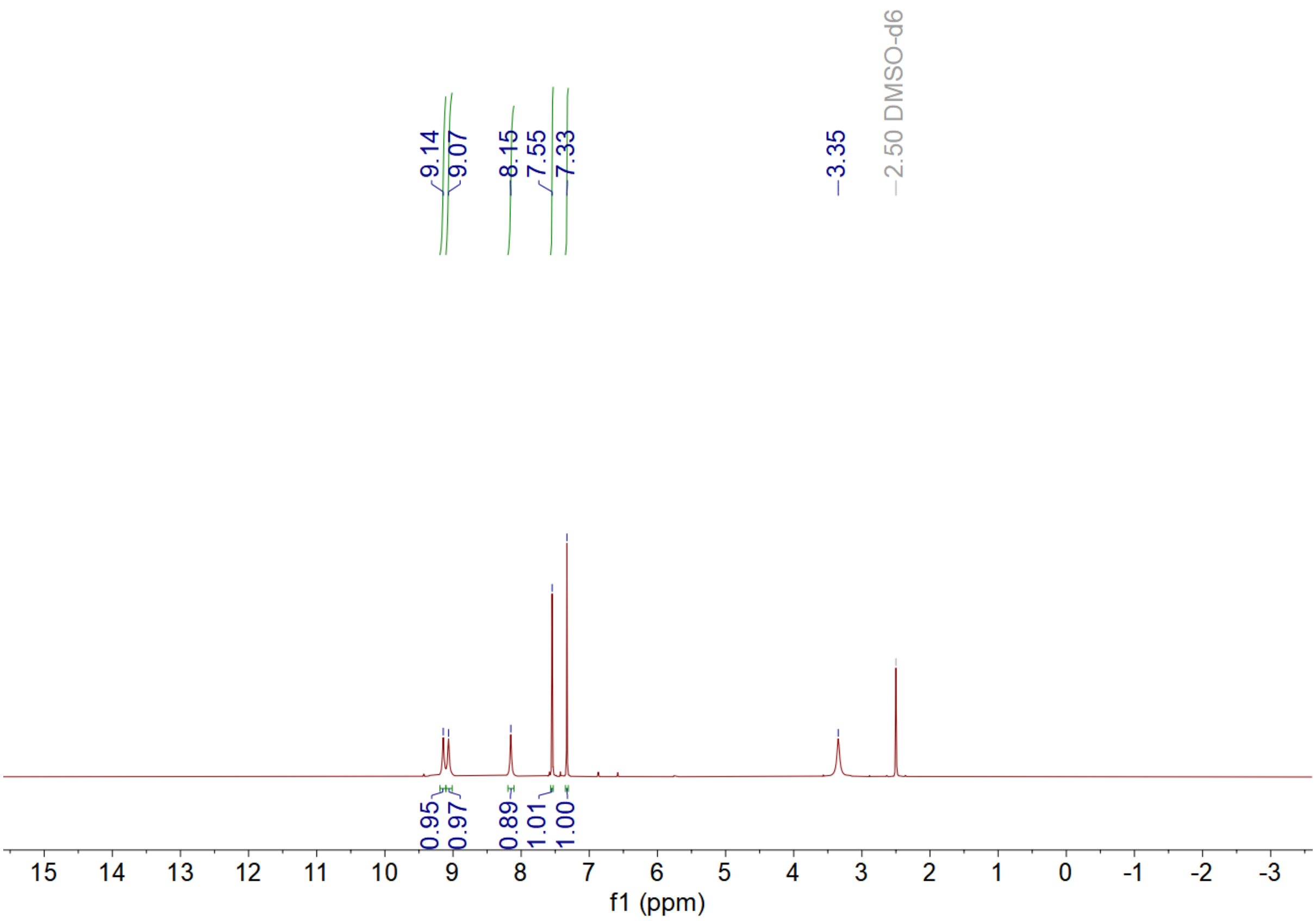

Figure S1. ^{1}H-NMR spectrum of 2,3,6,7,9,10-hexahydroxyphenanthrene (**3**) (25 °C, 500 MHz, DMSO-d6).

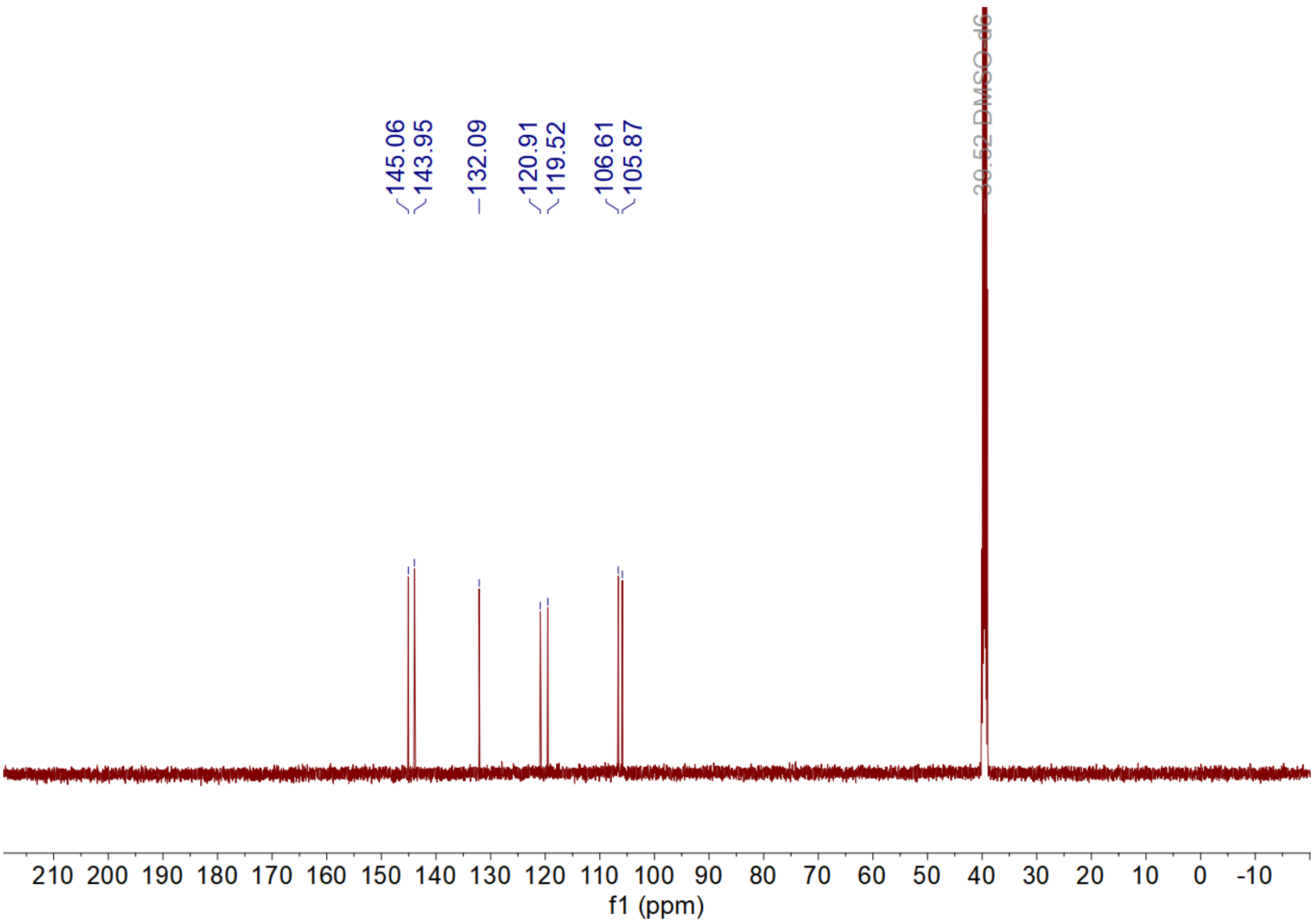

Figure S2. ^{13}C-NMR spectrum of 2,3,6,7,9,10-hexahydroxyphenanthrene (**3**) (25 °C, 500 MHz, DMSO-d6).

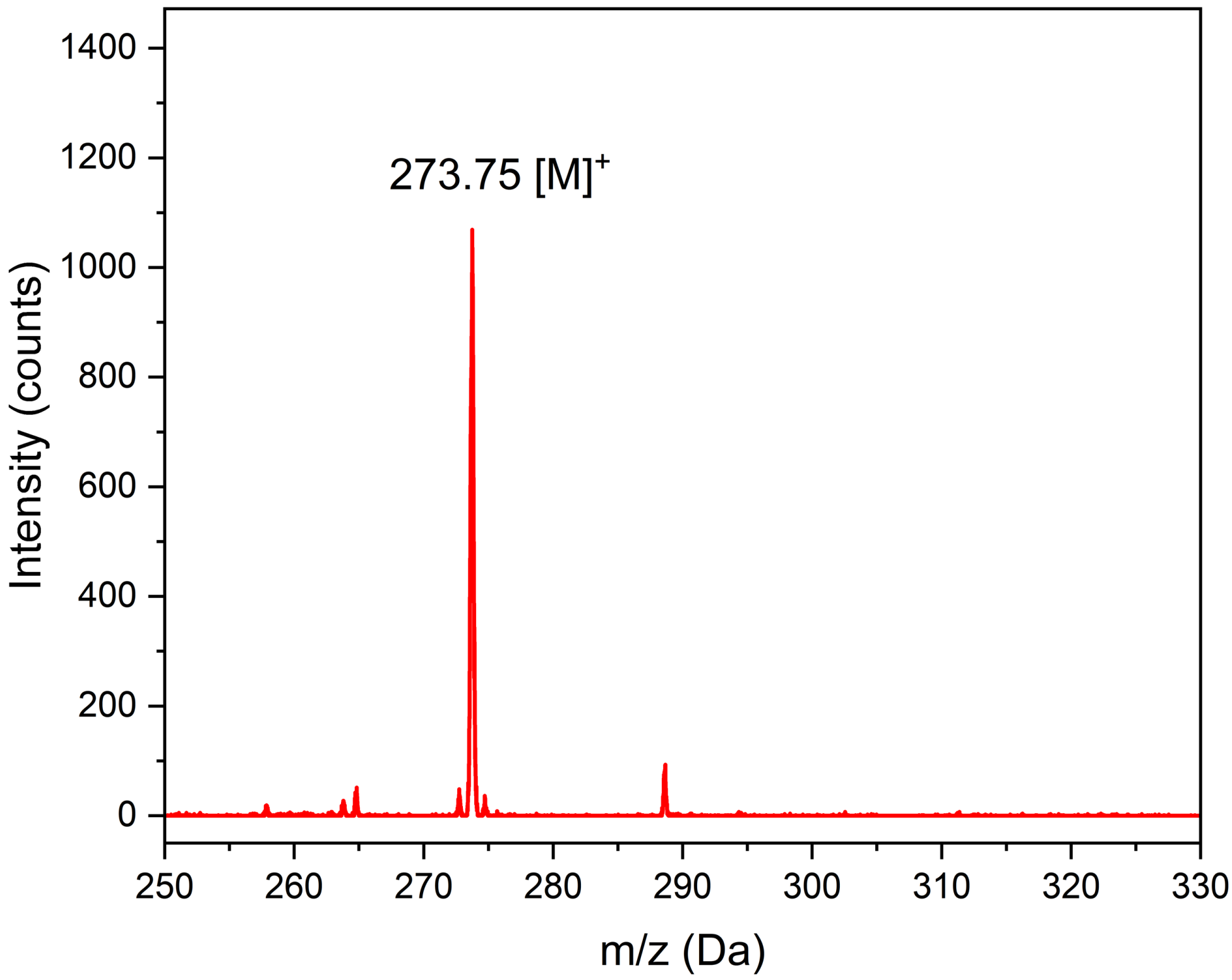

Figure S3. MALDI ToF of 2,3,6,7,9,10-hexahydroxyphenanthrene (**3**).

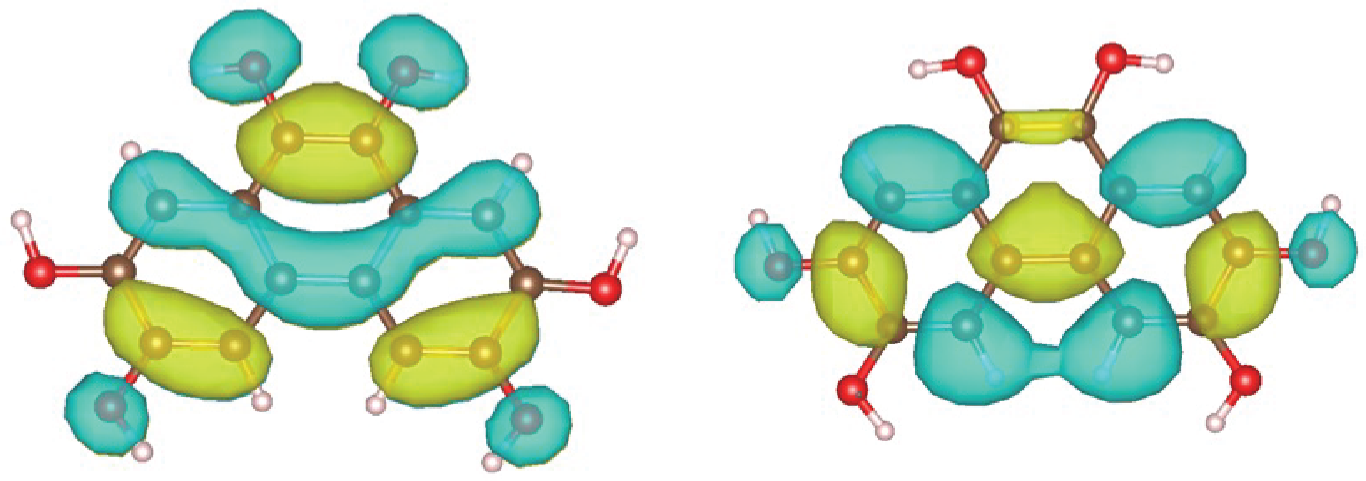

HOMO (−5.06 eV)   LUMO (−1.03 eV)

Figure S4. HOMO and LUMO of HHP, and the corresponding orbital energies (B3LYP/def2-SVP).

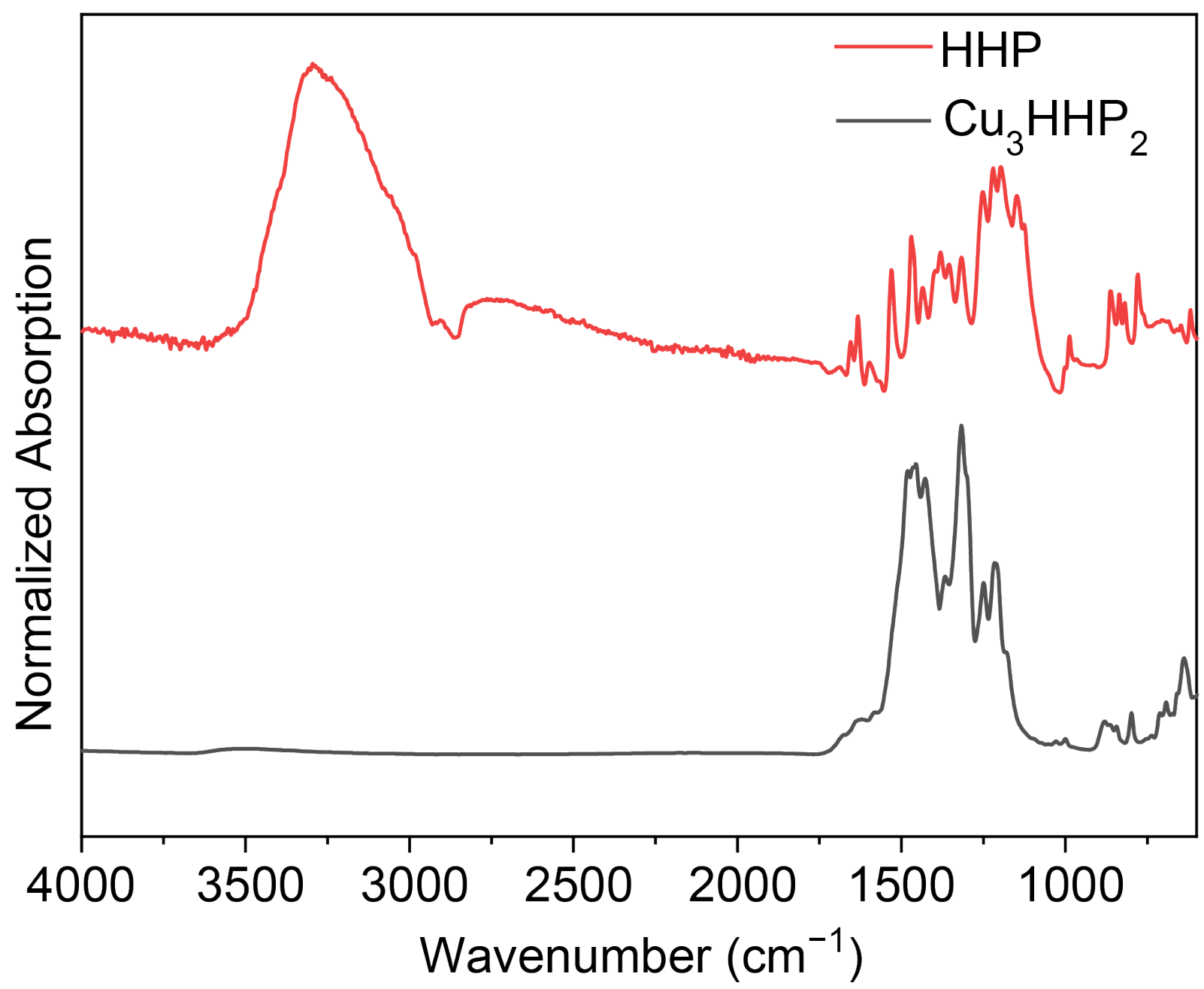

Figure S5. ATR-IR spectra of $Cu_3(HHP)_2$ (red) and the HHP linker (black).

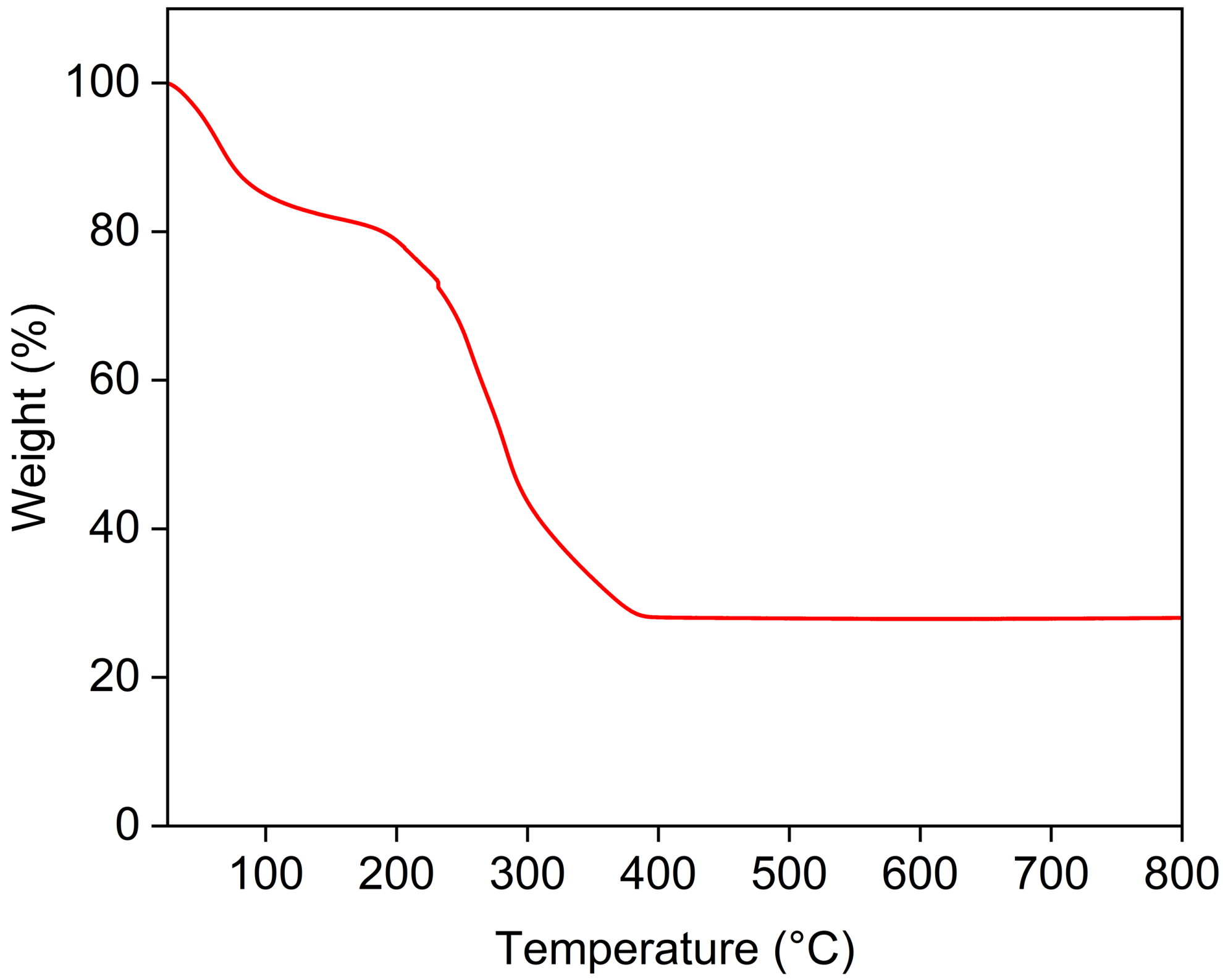

Figure S6. TGA traces of $Cu_3(HHP)_2$, collected at a heating rate of 2.0 °C/min under N_2 gas flow of 5 mL/min on a platinum pan from room temperature to 800 °C.

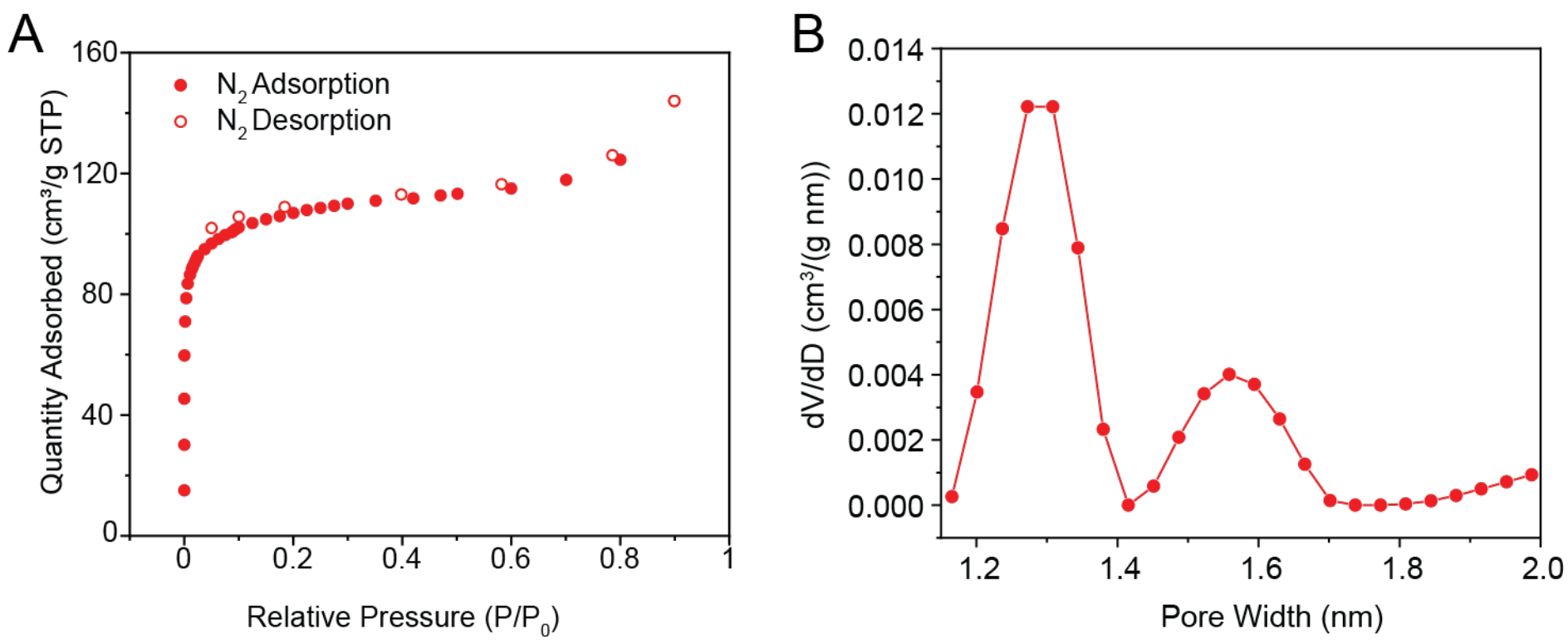

Figure S7. (A) Nitrogen adsorption isotherms for $Cu_3(HHP)_2$. The isotherm was fit to the BET equation to give an apparent BET surface area of 407 m²/g. The sample was degassed at 80 °C overnight before the measurement. (B) Pore size distribution derived from the N_2 sorption data, showing two pore-size populations centered at approximately 1.27 and 1.57 nm, consistent with the two distinct pore environments in the proposed structure.

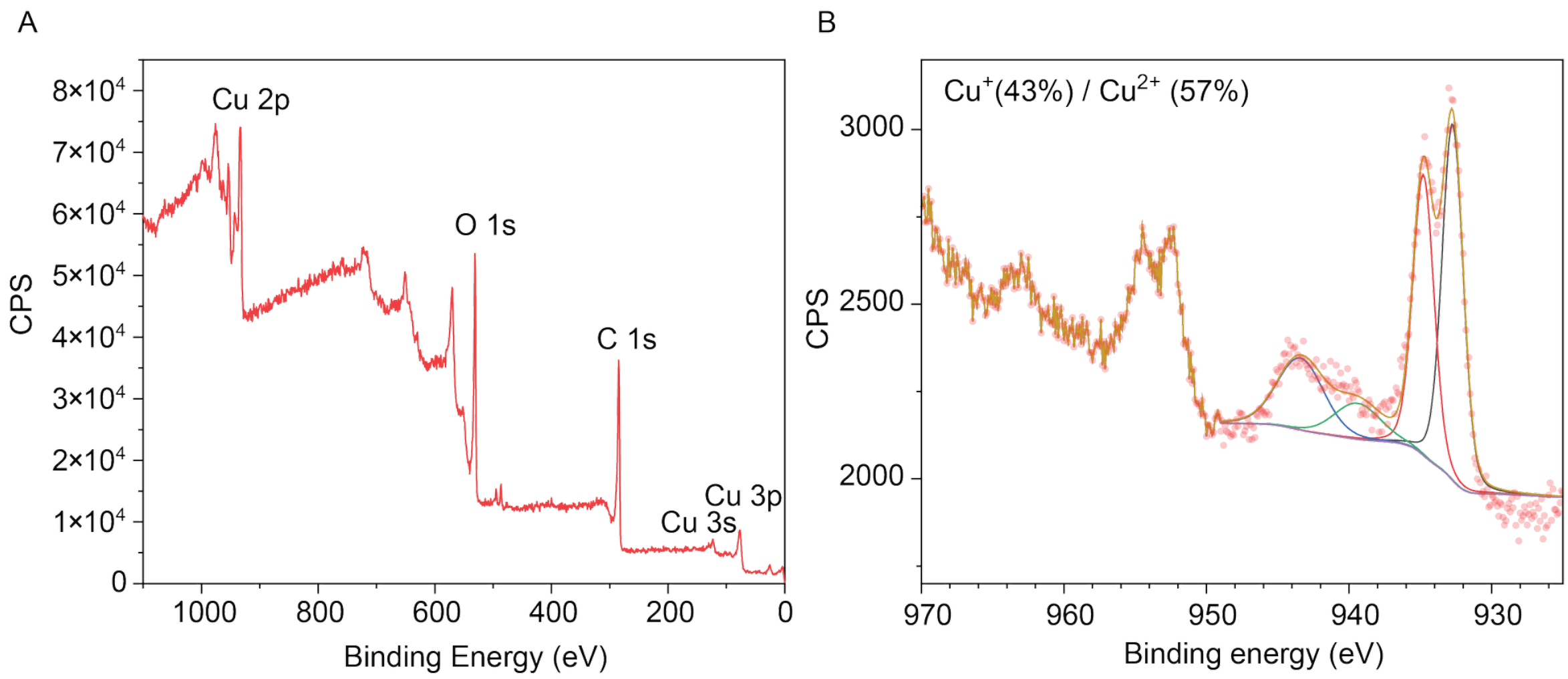

Figure S8. (A) XPS survey data of $Cu_3(HHP)_2$ (B) XPS spectra of $Cu_3(HHP)_2$ at Cu2p regions.

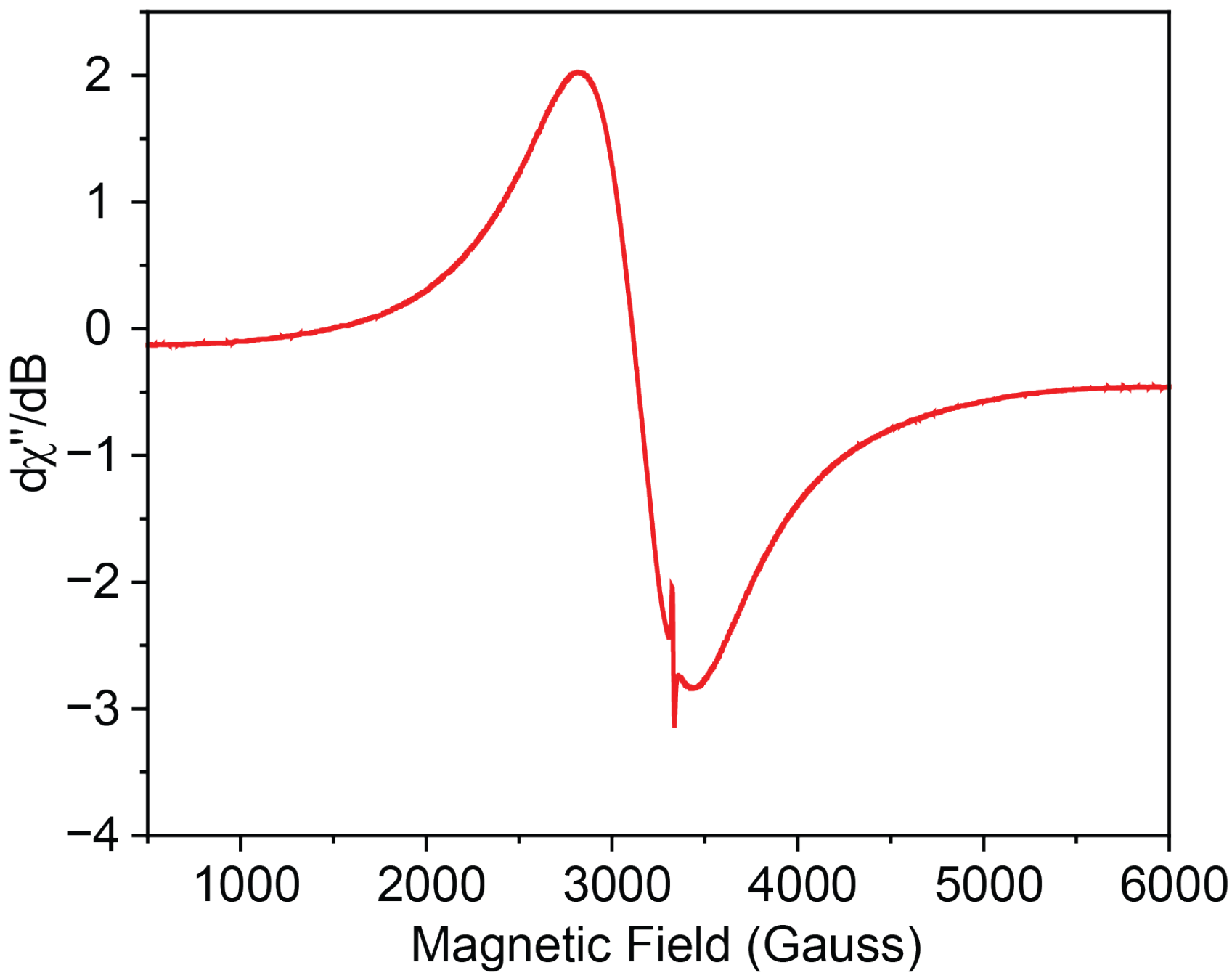

Figure S9 EPR spectra of $Cu_3(HHP)_2$ at room temperature.

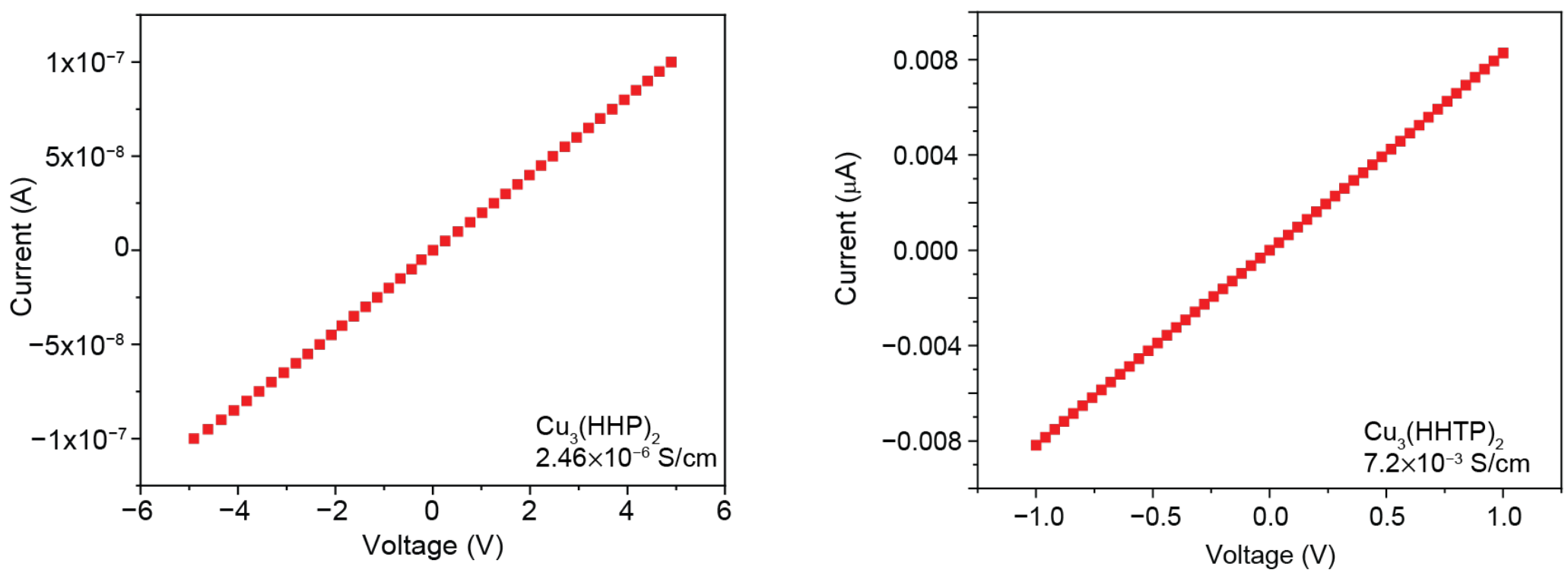

Figure S10. I-V curves of 4-probe devices of $Cu_3(HHP)_2$ and $Cu_3(HHTP)_2$ under the same conditions

Table S1. Summary of electronic conductivities (300 K) for 4-probe devices of $Cu_3(HHP)_2$. Samples are from multiple different batches. The measurements were done under the ambient conditions.

**Sample**	**Electrical conductivity (S/cm)**
Device 1	2.50×10^{-6}
Device 2	2.02×10^{-6}
Device 3	1.90×10^{-6}
Device 4	3.58×10^{-6}
Device 5	1.40×10^{-6}
Device 6	3.33×10^{-6}
**Average**	2.46×10^{-6}

Table S2. Reported conductivity value of $Cu_3(HHTP)_2$ powder

Method	Conductivity (S/cm)	Reference
Film (4-probe)	0.29	Angew. Chem. Int. Ed. 2020, 59, 1118 –1123
Film (2-probe)	0.02	Angew. Chem. Int. Ed. 2017, 56, 16510 –16514
Pellet (4-probe)	0.1	ACS Cent. Sci. 2019, 5, 12, 1959–1964
Pellet (4-probe)	0.045	CrystEngComm 2018, 20, 6458-6471
Pellet (4-probe)	0.02	Sensors 2017, 17, 2192.
Pellet (4-probe)	0.0076	Chem. Mater. 2016, 28, 15, 5264–5268
Pellet (4-probe)	0.008	J. Am. Chem. Soc. 2020, 142, 39, 16513–16517
Pellet (2-probe)	0.01	Nat Commun. 2019, 10, 4948
Pellet (2-probe)	0.003	Adv. Funct. Mater. 2017, 27, 1702067
Pellet (vdP)	0.027	ACS Nano 2019, 13, 6, 6711–6719

References

(1) Wang, J.; Chen, T.; Jeon, M.; Oppenheim, J. J.; Tan, B.; Kim, J.; Dincă, M. Superior Charge Transport in Ni-Diamine Conductive MOFs. *J. Am. Chem. Soc.* **2024**, *146* (29), 20500–20507. https://doi.org/10.1021/jacs.4c06935.

(2) Kresse, G.; Furthmüller, J. Efficient Iterative Schemes for Ab Initio Total-Energy Calculations Using a Plane-Wave Basis Set. *Phys. Rev. B* **1996**, *54* (16), 11169–11186. https://doi.org/10.1103/PhysRevB.54.11169.

(3) Jeon, M.; Kim, M.; Lee, J.-S.; Kim, H.; Choi, S.-J.; Moon, H. R.; Kim, J. Computational Prediction of Stacking Mode in Conductive Two-Dimensional Metal–Organic Frameworks: An Exploration of Chemical and Electrical Property Changes. *ACS Sens.* **2023**, *8* (8), 3068–3075. https://doi.org/10.1021/acssensors.3c00715.

(4) Blöchl, P. E. Projector Augmented-Wave Method. *Phys. Rev. B* **1994**, *50* (24), 17953–17979. https://doi.org/10.1103/PhysRevB.50.17953.

(5) Kresse, G.; Joubert, D. From Ultrasoft Pseudopotentials to the Projector Augmented-Wave Method. *Phys. Rev. B* **1999**, *59* (3), 1758–1775. https://doi.org/10.1103/PhysRevB.59.1758.

(6) Perdew, J. P.; Burke, K.; Ernzerhof, M. Generalized Gradient Approximation Made Simple. *Phys. Rev. Lett.* **1996**, *77* (18), 3865–3868. https://doi.org/10.1103/PhysRevLett.77.3865.

(7) Grimme, S.; Ehrlich, S.; Goerigk, L. Effect of the Damping Function in Dispersion Corrected Density Functional Theory. *J. Comput. Chem.* **2011**, *32* (7), 1456–1465. https://doi.org/10.1002/jcc.21759.

(8) Wang, L.; Maxisch, T.; Ceder, G. Oxidation Energies of Transition Metal Oxides within the $\mathrm{GGA}+\mathrm{U}$ Framework. *Phys. Rev. B* **2006**, *73* (19), 195107. https://doi.org/10.1103/PhysRevB.73.195107.

(9) Butler, K. T.; Hendon, C. H.; Walsh, A. Electronic Chemical Potentials of Porous Metal–Organic Frameworks. *J. Am. Chem. Soc.* **2014**, *136* (7), 2703–2706. https://doi.org/10.1021/ja4110073.

(10) Trosien, S.; Waldvogel, S. R. Synthesis of Highly Functionalized 9,10-Phenanthrenequinones by Oxidative Coupling Using MoCl5. *Org. Lett.* **2012**, *14* (12), 2976–2979. https://doi.org/10.1021/ol300948u.
